# lncRNA CRNDE Affects Th17/IL-17A and Inhibits Epithelial-Mesenchymal Transition in Lung Epithelial Cells Reducing Asthma Signs

**DOI:** 10.1155/2023/2092184

**Published:** 2023-01-27

**Authors:** Yu Yuan, Yi He, Binaya Wasti, Wentao Duan, Jingsi Jia, Zhifeng Chen, Xudong Xiang, Qingping Zeng

**Affiliations:** ^1^Department of Respiratory Medicine, The Second Xiangya Hospital, Central South University, Changsha, Hunan 410011, China; ^2^Department of Emergency, The Second Xiangya Hospital, Central South University, Changsha, Hunan 410011, China; ^3^Department of Respiratory and Critical Care Medicine, Longshan County People's Hospital, Longshan, Hunan 416899, China

## Abstract

**Background:**

Asthma treatment is difficult due to disease heterogeneity and comorbidities. In addition, the development of drugs targeting the underlying mechanisms of asthma remains slow. We planned to identify the most upregulated differentially expressed long noncoding RNA in asthma to explore its regulatory patterns and pathways in asthma.

**Methods:**

We sensitized mice using a mixture of ovalbumin, house dust mites, and lipopolysaccharide to establish an asthma mouse model. We also sensitized asthma cells with TGF-*β*1 in an *in vitro* model. We performed a microarray analysis to identify the lncRNA with the differential expression level in model mice. We applied hematoxylin and eosin and Masson's trichrome stainings to mouse tissues to quantify the tissue damage extent. Next, we assess the levels of lncRNA CRNDE, miR-29a-3p, TGF-*β*1, MCL-1, E-cadherin, vimentin, and snail. We counted the percentages of Th17 cells using flow cytometry. Finally, we performed a dual-luciferase reporter assay to assess the association between lncRNA CRNDE and miR-29a-3p.

**Results:**

We successfully established asthma mouse/cell models and selected the lncRNA CRNDE for our study. Transfection of si-CRNDE reduced the degree of injury and inflammation in the mouse model and reversed the TGF-*β*1-induced epithelial-mesenchymal transition (EMT) in the cell model. Moreover, the E-cadherin level was upregulated, and the levels of IL-17A, vimentin, snail, and *α*-SMA were downregulated. We also discovered that lncRNA CRNDE negatively regulated miR-29a-3p and that this one in turn inhibited MCL-1 in mice. After lncRNA CRNDE expression downregulation, the level of miR-29a-3p was increased, and we detected reduced levels of MCL-1 and EMTs.

**Conclusions:**

lncRNA CRNDE expression downregulation led to reduced inflammation and reduced lung damage in mice with induced asthma, it inhibited the EMTs of lung epithelial cells via the miR-29a-3p/MCL-1 pathway, and it reduced the levels of Th17/IL-17A cells to reduce asthma signs.

## 1. Introduction

Asthma is a chronic lung inflammatory disease resulting from complex and heterogeneous gene-environment interactions [1]. Its main symptoms include recurrent wheezing and airflow obstruction [2]. Exposure to air pollution, allergens, intake of acetaminophen, and polyunsaturated fatty acids have all been found to cause asthma exacerbations [3]. Asthma affects an estimated 339 million people worldwide, and 5–10% of them present symptoms that are not adequately controlled with the current treatments [4]. The management of asthma is difficult due to disease heterogeneity, comorbidities, and care system complexity [5]. Approximately 130 genetic loci have been identified in asthma-related genetic studies, but the development of drugs targeting the underlying mechanisms of asthma remains slow [4].

T helper cells (Th) participate in the pathogenesis of asthma [6]. Clinically, asthma has been divided into allergic and nonallergic subtypes [1]. In-depth studies on the pathogenesis of severe and refractory asthma have shown that airway neutrophil inflammation and airway remodeling are important causes of severe and refractory asthma. Th17 cells are involved in neutrophil inflammation and airway remodeling in severe asthma [7, 8]. Interleukin 17A (IL-17A) produced by Th17 cells can stimulate neutrophil airway inflammation [9] and is associated with increased airway hyperresponsiveness [10–12]. Moreover, IL-17A promotes lung inflammation and fibrosis of lung epithelial cells [13] and might enhance the occurrence of transforming growth factor-beta1- (TGF-*β*1-) induced epithelial-mesenchymal transition (EMT) in pulmonary epithelial cells [14]. These results suggest that the Th17/IL-17A cell/cytokine pair is closely associated with EMT in asthma.

Airway EMTs are important during airway remodeling in asthma, and the process affects the asthma barrier [15] leading to impaired epithelial barrier function, extracellular matrix deposition, and decreased lung function [16]. EMT of the bronchial epithelium is induced by miR-23b-3p [17]. EMT in asthmatic airway repair is also regulated by the IL-33/CD146 axis [15]. Gene-level EMT regulation has been trending as a target for new asthma treatments.

Long noncoding RNAs (lncRNA) can regulate gene expression at epigenetic transcription and translation sites [18], and many lncRNAs have a role in various dynamic disease processes [19]. Studies have shown that lncRNAs are associated with EMT [20, 21]. For example, the MALAT1 lncRNA is involved in the EMT of human HaCaT cells [22]; the SNHG6 lncRNA activates the TGF-*β* pathway and regulates EMT to promote the development of colorectal cancer [23]. Tumour exosomes promote Th17 cell differentiation in colorectal cancer by expressing the CRNDE-h lncRNA [24]. The lncRNA CRNDE has also been shown to delay the encephalopathy worsening after carbon monoxide poisoning [25]. In addition, the same lncRNA reduced the chemical resistance of gastric cancer through SRSF6-regulated PICALM variable splicing [26].

Research for the development of drugs targeting pathogenic mechanisms of asthma faces many challenges. Herein, we planned to select the most upregulated differentially expressed lncRNA in mice with induced asthma through microarray analysis to explore its regulation and asthma mechanisms. Although we are limited by time and budget constraints, we still believe that our results provide a valuable preliminary assessment of the lncRNA CRNDE as a target for new asthma treatments.

## 2. Materials and Methods

### 2.1. Materials

We used C57BL/6 mice (Shanghai Slac Laboratory Animal, Shanghai, China) in our experiments. Human lung epithelial cells BEAS-2B (No. CRL-9609) were purchased from the American Type Culture Collection (Manassas, USA). The bronchial epithelial growth medium was purchased from BulletKit (Lonza, Anaheim, USA). The house dust mite (HDM) extract was purchased from GREER® (Lenoir, USA). The ovalbumin (OVA, A5503-1G), lipopolysaccharide (LPS, L2880), and aluminum hydroxide (Al(OH)_3_, 239186) were all obtained from Sigma-Aldrich (Merck KGaA, Germany). The ionomycin (S1672), phorbol myristate acetate (P21120), and red blood cell lysate (C3702) were produced by Beyotime (USA). The moenomycin (M8670) was obtained from Solarbio (China). The CD3 (DXT-130-109-877), CD4 (DXT-130-109-498), and IL-17 (130-103-448) antibodies were purchased from Miltenyi (USA). The fixed-film piercing agent (C03-03002) was obtained from Bioss (China). The CRNDE NC-lncRNA, CRNDE si-lncRNA, NC mimic, miR-29a-3p mimic, oe-NC, oe-MCL-1, pHG-miRTarget- MCL-1 WT-3U, and CRNDE pHG-miRTarget-lncRNA WT/mutant reporter plasmids were purchased from GenePharma (Shanghai, China). The primary antibodies (E-cadherin, 20874-1-AP; vimentin 10366-1-AP), coralite488-conjugated Affinipure Goat anti-rabbitlgg (H+L), and IL-17A (human, KE00203), IL-17A (mouse, KE10020), IL-10 (human, KE00170), IL-10 (mouse, KE10008), IL-6 (human, KE00139), and IL-6 (mouse, KE10007) for ELISA kits were obtained from Proteintech (USA). RIPA lysate (AWB0136) and 1x permeabilization buffer (AWI0603a/b) were obtained from Abiowell (China). The BCA protein kit (ab102536) was obtained from Abcam (UK). The TRIzol reagent (15596-026) was obtained from Invitrogen (USA). The mRNA reverse transcription kit (CW2569) was obtained from Kangwei Century (China). The Cell Stimulation Cocktail (00-4975-93) and Transcription Factor Staining Buffer (00-5523-00) were purchased from eBioscience (USA). The primer sequences of the *CRNDE* and *IFNGAS1* lncRNAs and of the E-cadherin, vimentin, snail, *α-SMA*, *MCL-1*, miR-29a-3p, miR-29b-3p, miR-29c-3p, miR-181a-5p, miR-181b-5p, and *GAPDH* genes were provided by Sangon Biotech (China). The RNeasy Mini Kit was obtained from Qiagen (Hilden, Germany). The NanoDrop ND-1000 was purchased from Nano Drop Technologies (USA). The Agilent DNA Microarray Scanner (G2505C) was obtained from Agilent Technologies (Amstelveen, Netherlands). The fluorescent quantitative PCR instrument (PIKOREAL96) was obtained from Thermo Fisher (USA). The flow cytometer (A00-1-1102) was obtained from Beckman (USA).

### 2.2. Animal Model [27]

The Institutional Animal Care and Use Committee (IACUC) of The Second Xiangya Hospital (Central South University) reviewed and approved the animal use protocol listed below (No. 2022001) on January 4 of 2022. We conducted experiments under the relevant institutional and national guidelines for the care and use of laboratory animals.

We divided C57BL/6 mice into control and model groups. We prepared a sensitization agent mixing 200 *μ*L of normal saline, 100 *μ*g of OVA, 100 *μ*g of HDM, and 15 *μ*g of LPS combined with 2 mg of Al(OH)_3_ hydrate. The mice were intraperitoneally injected with either sensitization agent or normal saline on days 0, 1, and 2. The details of experimental animal groups and corresponding treatments are shown in Table 1.

Next, we treated the mice in an atomizer for 30 minutes on days 14, 15, 18, and 19. The atomizer in the model group atomized a 6% ovalbumin (OVA) solution (diluted with normal saline). The atomizer in the control group was loaded with an equivalent amount of normal saline. We performed mouse euthanasia by cervical dislocation on day 21 [28].

### 2.3. Separation of TH17 Cells from the Spleen

We initially obtained a cell suspension from the fluid left after mouse spleen grinding. The cell precipitates were lyzed with red blood cell lysate at room temperature for 3 min and then centrifuged and suspended in 1640 medium containing 10% FBS. The cells were then stimulated with a Cell Stimulation Cocktail (plus protein transport inhibitors) and cultured for 4 h at 37°C. We used Transcription Factor Staining and 1x permeabilization buffers to treat the cells. After adding antibodies, we allowed interactions to occur for 30 minutes. The cells were cleaned with PBS and then detected by flow cytometry.

### 2.4. Culture and Treatment of BEAS-2B Cells

We cultured human lung epithelial cells, BEAS-2B [29], in bronchial epithelial growth medium BulletKit at 37°C in a 5% CO_2_ atmosphere. The cells were divided into 10 groups (Table 2). We subcultured the cells at a density of 1 × 10^5^/mL. After culturing the cells in the presence of 10 ng/mL TGF-*β*1 for 48 h, we transfected them with plasmid. The CRNDE NC-lncRNA, CRNDE si-lncRNA, NC mimic, miR-29a-3p mimic, oe-NC, and oe-MCL-1 shRNAs were supplied by GenePharma. We transferred BEAS-2B cells onto plates for transfections following the manufacturer's instructions.

### 2.5. Microarray Analysis

We checked the RNA quality using a NanoDrop ND-1000 analyzer. We analyzed lncRNA expression profiles of lung tissues using an Arraystar Mouse lncRNA Microarray V4.0. We followed the Agilent One-Color Microarray-Based Gene Expression Analysis protocol to label the samples and conduct array hybridizations. The labeled cRNAs were purified using the RNeasy Mini Kit. We measured the concentration and specific activity of the labeled cRNAs in a NanoDrop ND-1000 analyzer. The labeled cRNA was fragmented after mixing the blocking agent and fragmentation buffer. After heating the cRNA solution at 60°C for 30 min, we added GE hybridization buffer to dilute the labeled cRNA. We loaded the hybridization solution onto the lncRNA expression microarray slide. Finally, the hybridized arrays were washed, fixed, and scanned using the Agilent DNA Microarray Scanner.

We used the Agilent Feature Extraction software (version 11.0.1.1) to analyze the acquired images. Quantile normalization and subsequent data processing were performed with the GeneSpring GX v12.1 software package. Next, we filtered *P* values and fold changes to identify highly expressed lncRNAs with statistically significant differences between the control and model groups [30].

### 2.6. Hematoxylin and Eosin (HE) Staining

We harvested mice's lungs and fixed them in 4% paraformaldehyde. We embedded left lung specimens in paraffin and cut them into 4 *μ*m thick slices. After 12 h at 60°C, we soaked the paraffin slices in xylene three times. Next, we dehydrated the slices by submerging them in different concentrations of ethanol. We cleaned the slices with distilled water and stained them with hematoxylin for 10 minutes before treating them with PBS reverse blue. Next, we stained the slides with eosin for 4 minutes. The slices were dehydrated again and treated with xylene for 20 minutes. Finally, we sealed the slices with neutral resin and observed them under a microscope.

### 2.7. Masson's Trichrome Staining

We harvested the mice's lungs and fixed them in 4% paraformaldehyde. We embedded left lung specimens in paraffin and cut them into 4 *μ*m thick slices. After keeping them at 60°C for 12 h, we dehydrated them. The slices were then dyed in a nuclear dye solution for 30 s. After completely washing off the staining solution, we soaked the slices in distilled water and immersed them in ammonia for 10 minutes to reverse the blue nuclei. After simply removing the water on the surface of the slices, we added an appropriate amount of stain slurry for 5 minutes. Next, we used a rinse solution to clean the slices and added a separation solution to cover the slices for 30 seconds. The final washes were done with anhydrous ethanol after staining for 7 minutes. Finally, we treated the slices with xylene, sealed them with neutral resin, and observed them under a microscope.

### 2.8. Periodic Acid-Schiff (PAS) Staining

We harvested mice's lungs and fixed them in 4% paraformaldehyde. We embedded left lung specimens in paraffin and cut them into 4 *μ*m thick slices. After 12 h at 60°C, we soaked the paraffin slices in xylene three times. Next, we dehydrated the slices in solutions consisting of different concentrations of ethanol. After cleaning the slices with distilled water, we coated them with periodic acid and kept them immersed for 7 minutes. Then, we washed off the residual liquid and stained the slices with Schiff's solution for 4 minutes. Next, we further stained the slices with hematoxylin and treated them with PBS reverse blue. The slices were dehydrated again and treated with xylene for 20 minutes. Finally, we sealed the slices with neutral resin and observed them under a microscope.

### 2.9. Enzyme-Linked Immunosorbent Assay (ELISA)

We centrifuged the mice serum, alveolar lavage fluid, and cell supernatants at 1000 g for 15 minutes at 4°C and collected the supernatants for detection. We used IL-17A (human, KE00203), IL-17A (mouse, KE10020), IL-10 (human, KE00170), IL-10 (mouse, KE10008), IL-6 (human, KE00139), and IL-6 (mouse, KE10007) ELISA kits to measure relative levels of factors in samples.

### 2.10. White Blood Cell (WBC) Counts in Bronchoalveolar Lavage

After performing mouse euthanasia by cervical disruption on day 21 [28], we inserted a catheter into the mouse lungs through the trachea and added 1 mL of PBS to obtain lavage samples. We used a blood cell counter to assess the WBC density in bronchoalveolar lavage fluid.

### 2.11. Immunofluorescence (IF)

We used PBS to wash the tissue sections for 30 minutes and 4% paraformaldehyde to hold them in place. After 30 min of Triton treatment, we rinsed the sections with PBS. Finally, we sealed them with 5% BAS solution for 1 h. Primary antibodies (E-cadherin, vimentin, and coralite488-conjugated Affinipure Goat anti-rabbit (H+L)) were continually added to incubate for 1.5 h. We used a DAPI solution for 10 min to reveal the nuclei. Finally, we sealed the slices with glycerin and examined them under a fluorescence microscope.

### 2.12. Dual-Luciferase Reporter Assay

We seeded BEAS-2B cells into 24-well plates after 24 h of incubation. pHG-miRTarget-MCL-1 WT-3U and pHG-miRTarget-lncRNA CRNDE WT/mutant reporter plasmids were purchased from GenePharma. Following the manufacturer's instructions, we transiently cotransfected the BEAS-2B cells with miR-29a-3p mimic or NC mimic and 0.1 *μ*g of WT-3 U pHG-miRTarget-MCL-1 or CRNDE pHG-miRTarget-lncRNA (WT or mutant) reporter plasmids using Lipofectamine 2000. After 48 h, we measured the luciferase activity of firefly using a Dual-Luciferase Reporter Assay System. Finally, we recorded the results in a GloMax 20/20 Microplate Luminometer.

### 2.13. Western Blot

We mixed human chondrocytes with RIPA lysis buffer at low temperatures and measured protein contents using a bicinchoninic acid (BCA) protein kit. We ran denatured protein electrophoresis gels until the marker dye reached the bottom. Next, we sandwiched the gel in transfer buffer solution with filter paper and an NC membrane. The membrane was transferred at a constant current. PBST (5% skimmed milk) powder was applied to block the membrane for 1.5 h. Next, we added the first and secondary antibodies to the membrane in solution. We used a chemiluminescence imaging system to relatively quantify the protein bands on the membrane on the basis of the *β*-actin (internal reference) normalization. Supplementary Table 1 shows antibody information.

### 2.14. Quantitative Real-Time Polymerase Chain Reaction (qRT-PCR)

We extracted total RNA from samples using TRIzol reagent and used an mRNA reverse transcription kit to obtain cDNA. The primer sequences of *lncRNA CRNDE*, *IFNGAS1* lncRNA, *E-cadherin*, *vimentin*, *snail*, *α-SMA*, *MCL-1*, miR-29a-3p, miR-29b-3p, miR-29c-3p, miR-181a-5p, miR-181b-5p, and *GAPDH* genes were provided by Sangon Biotech (Supplemental Table 2). We analyzed DNA amplification and detection results using a fluorescent quantitative PCR instrument. We applied the 2^-△△CT^ method to assess relative mRNA levels with *GAPDH* as the reference gene.

### 2.15. Statistical Analysis

We used GraphPad Prism 9 for statistical analyses representing the data as means ± standard deviations ($\overset{¯}{X}\pm \text{SD}$). We applied Student's *t*-test to analyze differences between the two groups and one-way analysis of variance (ANOVA) to compare the differences between multiple groups. We calculated Pearson's correlation coefficients to detect associations between the studied variables. We considered *P* values < 0.05 as statistically significant.

## 3. Results

### 3.1. Asthma Mouse Model Established Successfully

**Figure 1:**
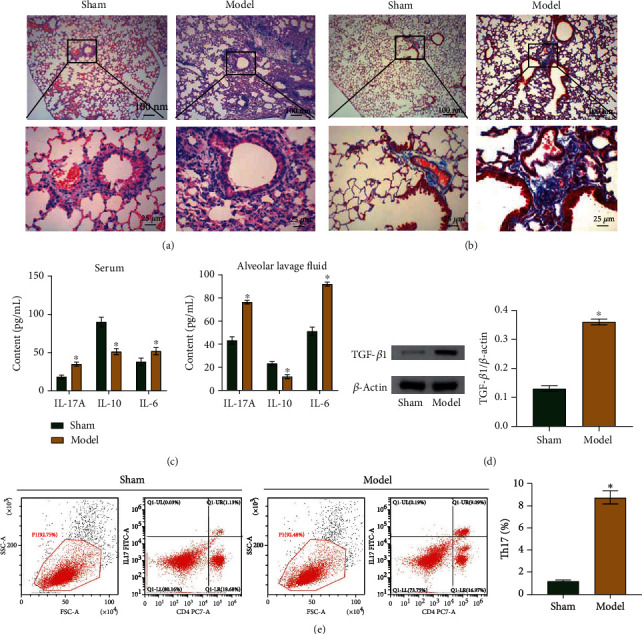
Asthma mouse model established successfully. (a) HE stained tissue samples to observe morphological changes in model mice. Observe the presence of inflammatory cell infiltration in the lung tissues of the model group. Scale bar = 25/100 *μ*m. (b) Masson's staining of samples to show the extent of tissue damage in model mice. The content of collagen fibers increased in the lung tissues of the model group. Scale bar = 25/100 *μ*m. (c) ELISA results showing levels of inflammatory factors. (d) Western blot showing TGF-*β*1 content. (e) Flow cytometry results showing percentages of Th17 cells. ^∗^*P* < 0.05 vs. control.

First, we established an asthma model in mice. Figures 1(a) and 1(b) show inflammatory cell infiltrations and increased collagen fiber content in lung tissues of the model group mice. In addition, the levels of inflammation factors, IL-17A and IL-6, were significantly higher while the IL-10 level was significantly lower in the model group compared to the levels in the control group (Figure 1(c)). Moreover, the model group mice presented higher levels of TGF-*β*1 than their counterparts (Figure 1(d)), and the percentage of Th17 cells in the spleens of model mice were also higher (Figure 1(e)). These results confirm the successful establishment of our asthma model.

### 3.2. Microarray Analysis of lncRNAs and Result Validation

**Figure 2:**
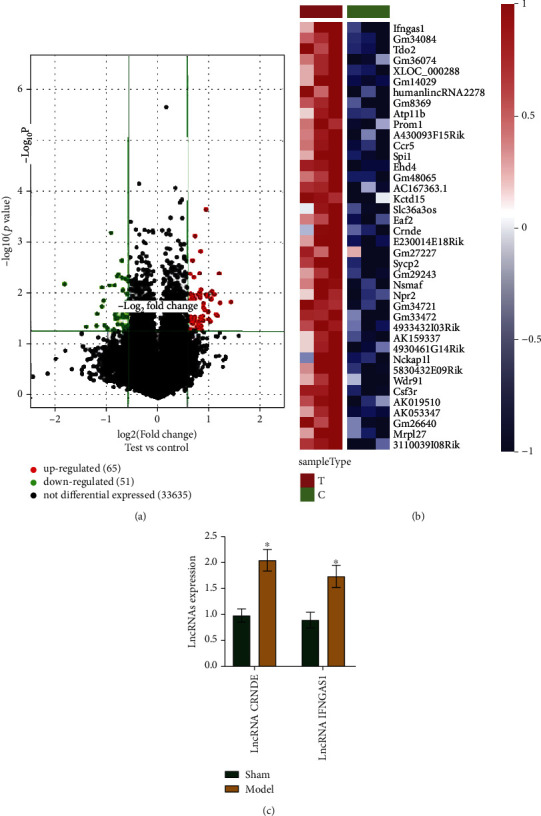
Microarray analysis of lncRNAs and results validation. (a) Volcano plot of lncRNAs. Genes in the upper left and right quadrants were significantly differentially expressed. (b) The heat map illustrates the 40 most upregulated genes. (c) qRT-PCR results showing the levels of CRNDE and IFNGAS1 lncRNAs. ^∗^*P* < 0.05 vs. control.

We used microarray analysis to identify lncRNA expressions associated with asthma. Our volcanic map results show that asthma changed the transcriptome significantly. We found 51 lncRNAs that were significantly downregulated in the model group and 65 lncRNAs that were significantly upregulated in the model group (Figure 2(a)). The heat map shows the 40 most upregulated genes (Figure 2(b)). We selected the most upregulated differentially expressed lncRNA CRNDE and IFNGAS1 in asthma for our follow-up experiments. After our qRT-PCR results to confirm the upregulation of the lncRNAs (Figure 2(c)), we selected the lncRNA CRNDE as the most upregulated differentially expressed lncRNA for the subsequent experiments.

### 3.3. CRNDE lncRNA Regulates EMT and Th17/IL-17A Expression in Mouse Lung Epithelial Cells

**Figure 3:**
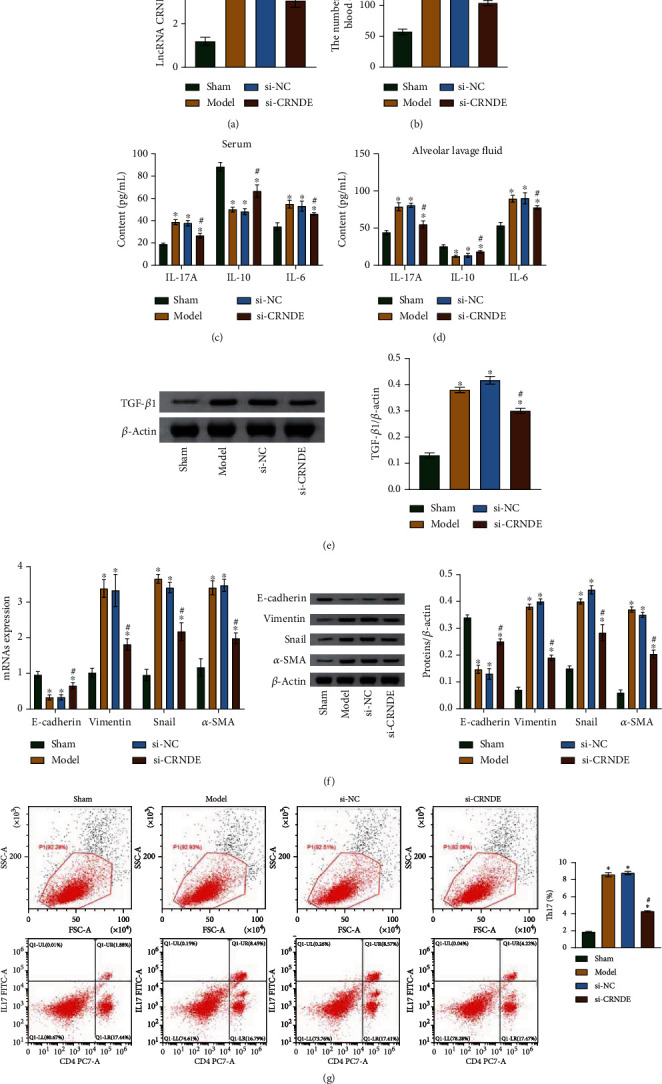
lncRNA CRNDE regulates EMT and Th17/IL-17A in mouse lung epithelial cells. (a) qRT-PCR results showing lncRNA CRNDE expression levels. (b) Total leucocyte counts in bronchoalveolar lavage. (c) ELISA was applied to detect levels of inflammatory factors. (d) ELISA was applied to detect levels of inflammatory factors. (e) Western blot results showing relative TGF-*β*1 content. (f) qRT-PCR and western blot results showing mRNA and protein expression levels of E-cadherin, vimentin, snail, and *α*-SMA. (g) Flow cytometry results showing percentages of Th17 cells. ^∗^*P* < 0.05 vs. control. ^#^*P* < 0.05 vs. si-NC.

We explored the effects of lncRNA CRNDE in mouse lung epithelial cells *in vivo*. We first checked the levels of lncRNA CRNDE in a mouse model of asthma and found the level in the model group to be significantly higher than that in the control group. In addition, on the model group mice, the level of lncRNA CRNDE was lower after a tail vein si-CRNDE injection than after an si-NC injection, a result that confirmed the success of the viral vector transfections (Figure 3(a)). The number of WBCs in the model group was higher than that in the control group, and the number of WBCs in the si-CRNDE group was lower than in the si-NC group (Figure 3(b)). According to the results of HE, PAS, and Masson's stainings, the inflammatory cell infiltrations, the numbers of PAS positive cells, and the collagen fiber contents were all increased in the model group. All these changes were reversed after transfection with si-CRNDE (Supplementary Figure S1).

The results of measurement of inflammatory factors in mice's serum or alveolar lavage fluid showed that IL-17A and IL-6 levels were significantly decreased and IL-10 levels were significantly increased after transfections with si-CRNDE (Figures 3(c) and 3(d)). Meanwhile, the levels of TGF-*β*1, vimentin, snail, and *α*-SMA were downregulated, and that of E-cadherin was increased (Figures 3(e) and 3(f)), and the percentages of spleen Th17 cells were decreased (Figure 3(g)). These results indicate that lncRNA CRNDE regulates EMT and Th17/IL-17A in mouse lung epithelial cells.

### 3.4. CRNDE lncRNA Overexpression Inhibits EMTs in Human Lung Epithelial Cells

**Figure 4:**
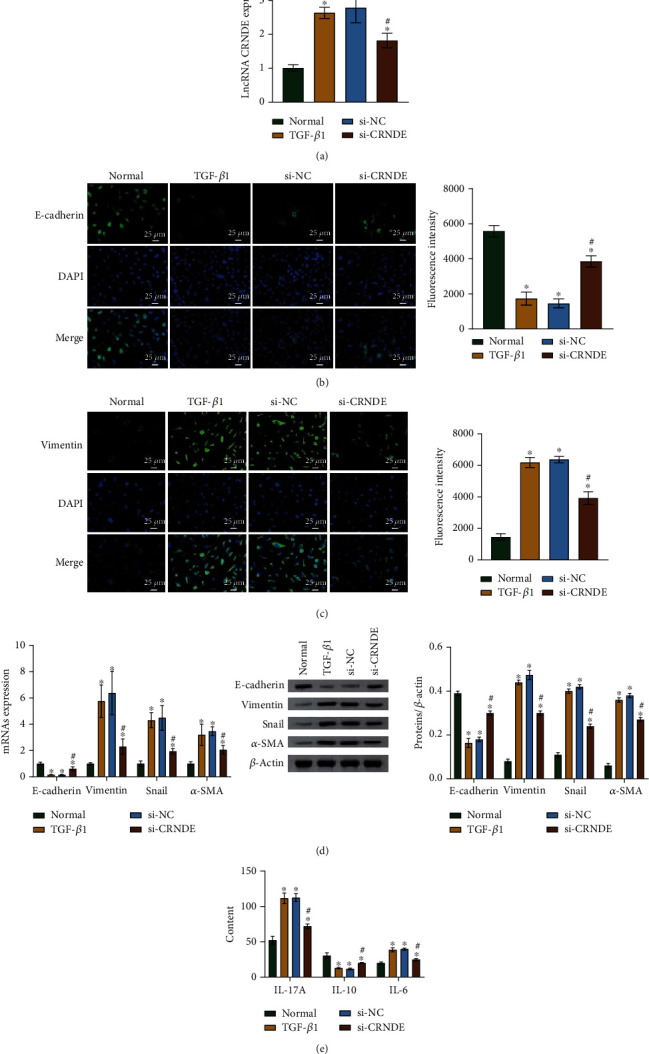
The CRNDE lncRNA inhibits EMTs in human lung epithelial cells. (a) qRT-PCR results showing lncRNA CRNDE levels. (b) Immunofluorescence (IF) results showing E-cadherin distribution detection after treating cells with 10 ng/mL of TGF-*β*1 for 48 h. Scale bar = 25 *μ*m. (c) IF results showing vimentin distribution detection after treating cells with 10 ng/mL of TGF-*β*1 for 48 h. Scale bar = 25 *μ*m. (d) qRT-PCR and western blot results showing mRNA and relative protein levels of E-cadherin, vimentin, snail, and *α*-SMA. (e) ELISA results showing levels of inflammatory factors. ^∗^*P* < 0.05 vs. control. ^#^*P* < 0.05 vs. si-NC.

To explore the function of the lncRNA CRNDE in human lung epithelial cells *in vitro*, we incubated the cells with TGF-*β*1 and transfected them with lncRNA CRNDE. We found that the TGF-*β*1 treatment significantly upregulated the levels of lncRNA CRNDE. The lncRNA CRNDE level in the si-CRNDE group was considerably lower than the level in the si-NC group (Figure 4(a)). TGF-*β*1 treatment significantly downregulated the level of E-cadherin and upregulated the levels of vimentin, snail, and *α*-SMA. Transfection with si-CRNDE reversed the TGF-*β*1-induced transition, upregulating E-cadherin levels and downregulating vimentin, snail, and *α*-SMA levels (Figures 4(b)–4(e)). Thus, lncRNA CRNDE inhibited EMTs in lung epithelial cells.

### 3.5. CRNDE lncRNA Targets miR-29a-3p

**Figure 5:**
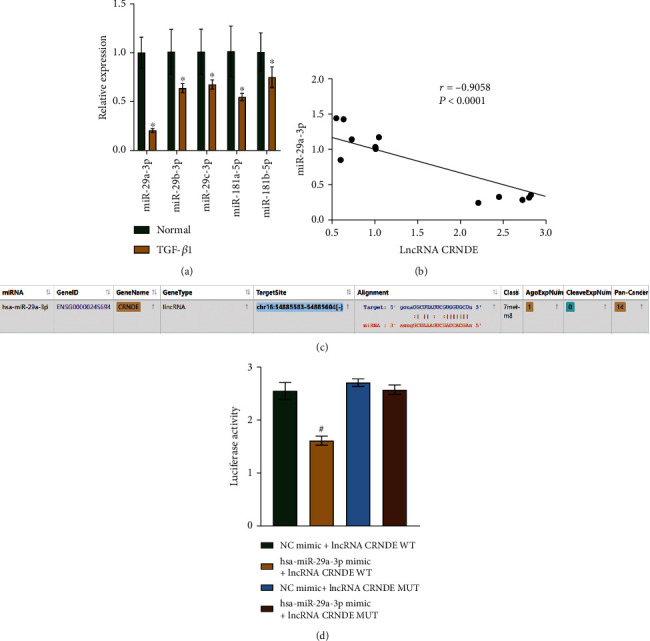
The lncRNA CRNDE targets miR-29a-3p. (a) qRT-PCR results showing the levels of miR-29a-3p, miR-29b-3p, miR-29c-3p, miR-181a-5p, and miR-181b-5p. (b) Pearson's correlation analysis of lncRNA CRNDE and miR-29a-3p expressions. (c) Target site prediction between lncRNA CRNDE and miR-29a-3p. (d) Dual-luciferase reporter assay showing an association between lncRNA CRNDE and miR-29a-3p. ^∗^*P* < 0.05 vs. control. ^#^*P* < 0.05*vs.* NC mimic+lncRNA CRNDE WT.

We searched for and selected the downstream target genes of the lncRNA CRNDE using miRDB and LncBase Predicted V.2 software. The models predicted and selected miR-29a-3p, miR-29b-3p, miR-29c-3p, miR-181a-5p, and miR-181b-5p as lncRNA CRNDE targets. Next, we performed qRT-PCR reactions to assess the levels of these target genes in mice treated with TGF-*β*1 and in controls, and we found that the difference in miR-29a-3p expressions between the two groups was the largest (Figure 5(a)). Moreover, miR-29a-3p was negatively correlated with the lncRNA CRNDE (Figure 5(b)). The starBase software predicted common targets between miR-29a-3p and lncRNA CRNDE (Figures 5(c) and 5(d)). Therefore, our results suggest that lncRNA CRNDE targets miR-29a-3p.

### 3.6. The lncRNA CRNDE Inhibits EMT via the miR-29a-3p/MCL-1 Axis in Human Lung Epithelial Cells

**Figure 6:**
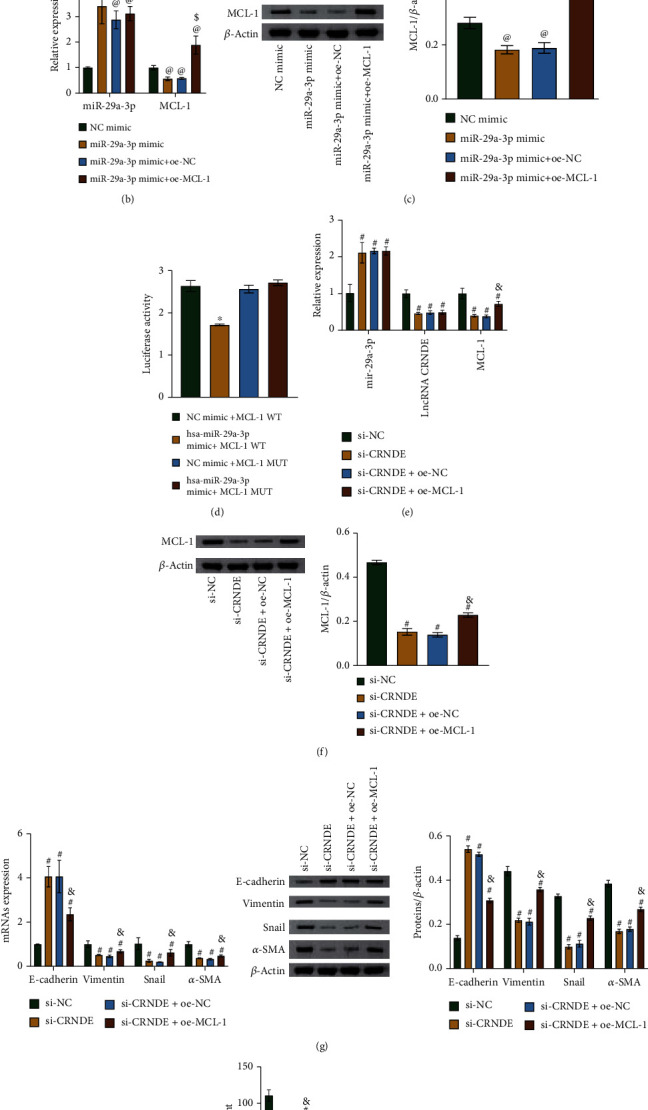
The lncRNA CRNDE inhibited EMT in human lung epithelial cells via the miR-29a-3p/MCL-1 axis. (a) Target site prediction between miR-29a-3p and MCL-1. (b) qRT-PCR results showing the mRNA levels of miR-29a-3p and MCL-1. (c) Western blot results showing the MCL-1 protein contents. (d) Dual-luciferase reporter assay results showing an association between miR-29a-3p and MCL-1. (e) qRT-PCR results showing the mRNA levels of miR-29a-3p, *lncRNA CRNDE*, and *MCL-1*. (f) Western blot results showing the MCL-1 protein abundances. (g) qRT-PCR and western blot results showing mRNA and protein levels of E-cadherin, vimentin, snail, and *α*-SMA. (h) ELISA results showing levels of inflammatory factors. ^@^*P* < 0.05 vs. NC mimic. ^$^*P* < 0.05 vs. miR-29a-3p mimic+oe-NC. ^∗^*P* < 0.05 vs. NC mimic+MCL-1 WT. ^#^*P* < 0.05 vs. si-NC. ^&^*P* < 0.05 vs. si-CRNDE+oe-NC.

After determining that lncRNA CRNDE targets miR-29a-3p, we used starBase to predict direct binding sites between miR-29a-3p and MCL-1 (Figure 6(a)), and then, we conducted experiments to validate the binding sites. Overexpression of miR-29a-3p increased the miR-29a-3p expression level and decreased the MCL-1 level. Overexpression of MCL-1 did not affect the miR-29a-3p level, but it increased the MCL-1 level (Figures 6(b) and 6(c)).

As shown in Figure 6(d), we found a functional association between miR-29a-3p and MCL-1. After transfection with si-CRNDE, the miR-29a-3p and E-cadherin levels increased, while the levels of lncRNA CRNDE, MCL-1, vimentin, snail, and *α*-SMA decreased (Figures 6(e)–6(g) and Supplementary Figure S2). After further transfections with oe-MCL-1, the levels of E-cadherin decreased, while the levels of MCL-1, vimentin, snail, and *α*-SMA increased (Figures 6(e) and 6(g) and Supplementary Figure S2). In addition, lncRNA CRNDE knockdown increased the IL-10 level and decreased the levels of IL-17A and IL-6 (Figure 6(h)). Overexpression of MCL-1 led to the opposite change in inflammatory factors (Figure 6(h)). Therefore, lncRNA CRNDE inhibited EMTs via the miR-29a-3p/MCL-1 axis in lung epithelial cells.

## 4. Discussion

First, we successfully constructed an asthma mouse model. We performed microarray analyses to select the most upregulated differentially expressed lncRNA, lncRNA CRNDE, for the subsequent part of the study. Next, we found that in lung epithelial cells of the model mice, lncRNA CRNDE regulated Th17/IL-17A, and in human lung epithelial cells, lncRNA CRNDE inhibited EMT via the miR-29a-3p/MCL-1 axis.

The most important asthma symptoms in patients include excessive mucus secretion, epithelial shedding, inflammatory cell infiltration, and others [31]. In the asthma mouse model, IL-17A induced increased airway neutrophil infiltration [32, 33]. The IL-6-induced inflammatory pathway increased the inflammatory response causing microvascular dilation and muscle contraction, two processes responsible for edema and airway narrowing during asthma attacks in humans [34, 35]. IL-10 is an anti-inflammatory mediator that protects the host against overreactions to pathogens and microbiota [36]. In our asthma mouse model sensitized with OVA, the lung tissues were significantly damaged and the contents of inflammatory cytokines (IL-17A and IL-6) were considerably increased, while the content of IL-10 was significantly decreased. The changes in inflammatory factors in the asthma model mice in our experiments are consistent with previous results. Thus, we successfully established a mouse model of asthma. Blocking the Th17 cell response has been shown to be beneficial as a therapeutic approach for asthma relief [37]. We also detected Th17 cell numbers in the spleen and found them to be significantly upregulated in the mice.

lncRNAs have roles in various dynamic disease processes [19]. lncRNAs are involved in gene regulation at posttranscriptional and translational levels in the cytoplasm, including through interactions with proteins in the cytoplasm [38] and regulating mRNA metabolism [39, 40]. With our microarray analysis results, we found 65 lncRNAs that were significantly upregulated in the model group. The upregulated genes included the CRNDE and IFNGAS1 lncRNAs. In children with asthma, lncRNA CRNDE is positively correlated with inflammatory cytokines and is a potentially useful prognostic marker for asthma management [41]. IFNGAS1 lncRNA has been associated with autoimmune diseases, including asthma [42]. We focused on lncRNA CRNDE due to its higher upregulated expression for the rest of the study. CRNDE lncRNA has been shown to trigger inflammation through the TLR3-NF-*κ*B cytokine signaling pathway [43]. In addition, lncRNA CRNDE overexpression has been shown to activate the TLR4/NF-*κ*B signaling pathway to accelerate LPS-induced inflammation and apoptosis of HK-2 cells via miR-146a regulation [44]. Moreover, the lncRNA CRNDE/miR-181a-5p/TLR4 axis has been shown to alter the pathogenesis of sepsis-associated inflammation [45]. In our heat map, we illustrate the 40 most upregulated lncRNAs genes of the 65 identified.

In our asthma model, a high lncRNA CRNDE expression was accompanied by a significant inflammatory response. Knock-down of lncRNA CRNDE reduced the lung injury caused by the asthma induction, increased the IL-10 content, and decreased the contents of IL-17A and IL-6 considerably while reducing the number of Th17 cells in the spleen.

TGF-*β*1 was also downregulated after lncRNA CRNDE knockdown. This may significantly inhibit Th lymphocyte activation and proliferation, limiting the effector functions that lead to destructive tissue responses [46]. Thus, lncRNA CRNDE could affect the immunological competence. After explaining the association between lncRNA CRNDE and the Th17/IL-17A relative factors, we further explored the association between the lncRNA CRNDE and EMTs. EMTs are known to progress under deficiency of the invasion-inhibitor E-cadherin [47] and upregulation of vimentin [48, 49], snail [50], and *α*-SMA [51]. Our results showed that transfection with si-CRNDE upregulated the E-cadherin levels and downregulated those of vimentin, snail, and *α*-SMA. Thus, lncRNA CRNDE inhibited EMTs.

**Figure 7:**
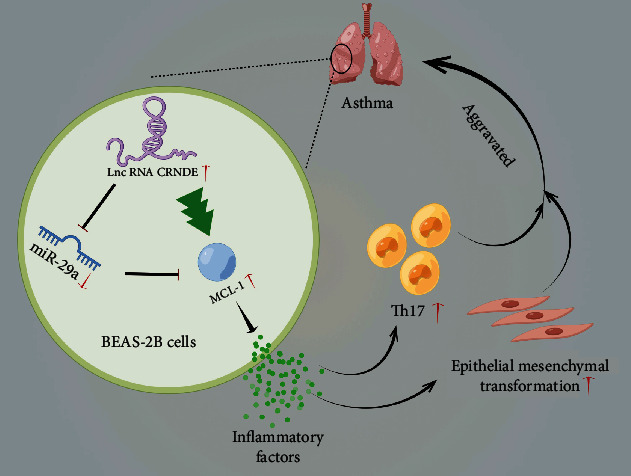
Figdraw graphical abstract showing the mechanism of lncRNA CRNDE on asthma (https://www.figdraw.com/static/index.html#/paint_about).

To further clarify the EMT inhibition mechanism of lncRNA CRNDE, we performed experiments with miR-29a-3p/MCL-1; our results show a stepwise regulatory association between the lncRNA CRNDE and the miR-29a-3p/MCL-1 axis. miR-29a-3p was negatively regulated by lncRNA CRNDE, and MCL-1 was negatively regulated by miR-29a-3p. After transfection with si-CRNDE, the levels of miR-29a-3p in cells were increased, and the levels of MCL-1 were decreased, while EMTs were inhibited. The negative regulatory effect of miR-29a-3p upon MCL-1 in peripheral blood has been documented [52]. miR-29a promoted apoptosis of intestinal epithelial cells in colitis by downregulating MCL-1 [53]. In our study, lncRNA CRNDE inhibited EMTs in lung epithelial cells via the downregulation of the miR-29a-3p/MCL-1 axis, which resulted in Th17/IL-17A effects that reduced asthma signs (Figure 7).

## 5. Limitations and Future Work

Our results open venues for future investigations. However, the database of asthmatic mice needs to be expanded to improve the accuracy of the results. We plan to search for regulators of lncRNA CRNDE by searching the databases of asthma comorbidities. We believe that the approach adopted in this study can be extended to include a screen on the basis of pulmonary heart disease, chronic respiratory failure, and other asthma complications to create a comprehensive prognostic toolkit for asthma and its complications.

## 6. Conclusions

lncRNA CRNDE expression was significantly upregulated in our asthma mouse model. In contrast, downregulation of lncRNA CRNDE in the lung epithelial cells of mice reduced asthma signs via Th17/IL-17A effects. After downregulating lncRNA CRNDE expression, the level of miR-29a-3p was increased, and the level of MCL-1 was decreased, while EMTs were inhibited. The lncRNA CRNDE inhibited EMT in lung epithelial cells via the miR-29a-3p/MCL-1 axis and affected Th17/IL-17A to reduce asthma signs. We plan to perform experiments to include expression data on comorbidities in the predictive database to be able to identify lncRNA CRNDE regulators.

## Figures and Tables

**Table 1:** Experimental animal groups and corresponding treatments.

Groups	Treatment			Abbreviation
	Injected intraperitoneally (200 *μ*L)	Atomization	Transfection	
1	Normal saline	Normal saline	—	Sham
2	Sensitization agent	6% OVA solution (diluted with normal saline)	—	Model
3			NC-lncRNA CRNDE	si-NC
4			si-lncRNA CRNDE	si-CRNDE

**Table 2:** Experimental cell groups and corresponding treatments.

Groups	Treatment		Abbreviation
	10 ng/mL TGF-*β*1	Transfection	
1	—	—	Normal
2	+	—	TGF-*β*1
3		NC-lncRNA CRNDE	si-NC
4		si-lncRNA CRNDE	si-CRNDE
5		NC mimic	NC mimic
6		miR-29a-3p mimic	miR-29a-3p mimic
7		miR-29a-3p mimic+oe-NC	miR-29a-3p mimic+oe-NC
8		miR-29a-3p mimic+oe-MCL-1	miR-29a-3p mimic+oe-MCL-1
9		si-lncRNA CRNDE+oe-NC	si-CRNDE+oe-NC
10		si-lncRNA CRNDE+oe-MCL-1	si-CRNDE+oe-MCL-1

## Data Availability

The data used to support the findings of this study are included within the article.

## Acronyms

Th cells: T helper cells
IL-17A: Interleukin 17A
TGF-*β*1: Transforming growth factor-beta1
EMT: Epithelial-mesenchymal transition
lncRNA: Long noncoding RNAs
IACUC: Institutional Animal Care and Use Committee
OVA: Ovalbumin
HE: Hematoxylin and eosin
PAS: Periodic acid-Schiff
ELISA: Enzyme-linked immunosorbent assay
WBCs: White blood cells
IF: Immunofluorescence
BCA: Bicinchoninic acid
qRT-PCR: Quantitative real-time polymerase chain reaction.

## Supplementary Materials

Supplementary Materials Supplementary Figure S1: HE, PAS, and Masson's stainings were applied to observe tissue morphologies. The model group samples displayed inflammatory cell infiltration; the number of PAS positive cells and the collagen fiber content were higher than those in the control group samples. These changes were reversed after transfection with si-CRNDE. Scale bar = 25/100 *μ*m. Supplementary Figure S2: IF was applied to detect the distribution of E-cadherin and vimentin. Scale bar = 25 *μ*m. ^∗^*P* < 0.05 vs. si-NC. ^#^*P* < 0.05 vs. si-CRNDE+oe-NC. Supplement table 1: the information of antibody. Supplement table 2: the primer sequence.
