# Supplementary material for: lncRNA CRNDE Affects Th17/IL-17A and Inhibits Epithelial-Mesenchymal Transition in Lung Epithelial Cells Reducing Asthma Signs
Source: Oxid Med Cell Longev. 2023 Jan 27;2023:2092184. doi: 10.1155/2023/2092184 (PMC9897922; doi:10.1155/2023/2092184)
Supplement: Supplementary Materials — Supplementary Figure S1: HE, PAS, and Masson's stainings were applied to observe tissue morphologies. The model group samples displayed inflammatory cell infiltration; the number of PAS positive cells and the collagen fiber content were higher than those in the control group samples. These changes were reversed after transfection with si-CRNDE. Scale bar = 25/100 μm. Supplementary Figure S2: IF was applied to detect the distribution of E-cadherin and vimentin. Scale bar = 25 μm. ∗P < 0.05 vs. si-NC. #P < 0.05 vs. si-CRNDE+oe-NC. Supplement table 1: the information of antibody. Supplement table 2: the primer sequence. [file 2092184.f1.zip › Supplement table 1. The information of antibody.docx]

**Supplement table 1. The information of antibody**

| Name | Article number | Source | Dilution rate | Molecular weight | Transfer film time | Company |
| --- | --- | --- | --- | --- | --- | --- |
| TGF-β1 | 21898-1-AP | Rabbit | 1:2000 | 44 KDa | 65 min | Proteintech (USA) |
| Vimentin | 10366-1-AP | Rabbit | 1:5000 | 54 KDa | 75 min | Proteintech (USA) |
| snail | 13099-1-AP | Rabbit | 1:500 | 29 KDa | 50 min | Proteintech (USA) |
| α-SMA | 55135-1-AP | Rabbit | 1:2000 | 42 KDa | 60 min | Proteintech (USA) |
| E-cadherin | 20874-1-AP | Rabbit | 1:5000 | 120-125 KDa | 150 min | Proteintech (USA) |
| MCL-1 | 16225-1-AP | Rabbit | 1:1000 | 35-40 KDa | 60 min | Proteintech (USA) |
| β-actin | 66009-1-Ig | Mouse | 1:5000 | 42 KDa | 60 min | Proteintech (USA) |
| HRP goat anti-Rabbit IgG | SA00001-2 | Rabbit | 1:6000 | / | 90 min | Proteintech (USA) |
| HRP goat anti-mouse IgG | SA00001-1 | Mouse | 1:5000 | / | 90 min | Proteintech (USA) |
