# Supplementary material for: lncRNA CRNDE Affects Th17/IL-17A and Inhibits Epithelial-Mesenchymal Transition in Lung Epithelial Cells Reducing Asthma Signs
Source: Oxid Med Cell Longev. 2023 Jan 27;2023:2092184. doi: 10.1155/2023/2092184 (PMC9897922; doi:10.1155/2023/2092184)
Supplement: Supplementary Materials — Supplementary Figure S1: HE, PAS, and Masson's stainings were applied to observe tissue morphologies. The model group samples displayed inflammatory cell infiltration; the number of PAS positive cells and the collagen fiber content were higher than those in the control group samples. These changes were reversed after transfection with si-CRNDE. Scale bar = 25/100 μm. Supplementary Figure S2: IF was applied to detect the distribution of E-cadherin and vimentin. Scale bar = 25 μm. ∗P < 0.05 vs. si-NC. #P < 0.05 vs. si-CRNDE+oe-NC. Supplement table 1: the information of antibody. Supplement table 2: the primer sequence. [file 2092184.f1.zip › Supplement table 2. The primer sequence..docx]

**Supplement table 2. The primer sequence.**

| Primer ID | 5’-3’ |
| --- | --- |
| H-GAPDH-F | ACAGCCTCAAGATCATCAGC |
| H-GAPDH-R | GGTCATGAGTCCTTCCACGAT |
| M-GAPDH-F | GCGACTTCAACAGCAACTCCC |
| M-GAPDH-R | CACCCTGTTGCTGTAGCCGTA |
| M-lncRNA CRNDE-F | CACAGTTCTTAACGGAGCCC |
| M-lncRNA CRNDE -R | CCTCCAAACATGACACCAGCA |
| M-lncRNA IFNGAS1-F | TAGGATGTGGGGATCTCGCT |
| M-lncRNA IFNGAS1-R | TATGCTGTCGTCTTGTGTGCT |
| M-E-cadherin-F | AGCCATTGCCAAGTACATCCTC |
| M-E-cadherin-R | CGCCTTCTGCAACGAATCCC |
| M-Vimentin-F | GTCCACACGCACCTACAGTCT |
| M-Vimentin-R | AAGTCCACCGAGTCTTGAAGC |
| M-snail-R | TGCTTTTGCTGACCGCTCCAAC |
| M-snail-R | GCACTGGTATCTCTTCACATCCGAGT |
| M-α-SMA-F | GCCCCTGAAGAGCATCCGAC |
| M-α-SMA-R | CCAGAGTCCAGCACAATACCAGT |
| H-lncRNA CRNDE-F | GGCGCTAACGGTCGGTAA |
| H-lncRNA CRNDE -R | ACGAGGGGACACGACTAGAG |
| H-lncRNA IFNGAS1-F | TGTGGGTCCAATGTGAAAAACAC |
| H-lncRNA IFNGAS1-R | TGTTAGCAGTTGGTGGGCTT |
| H-E-cadherin-F | ATTTTTCCCTCGACACCCGAT |
| H-E-cadherin-R | TCCCAGGCGTAGACCAAGA |
| H-Vimentin-F | CCCTTGACATTGAGATTGCCACC |
| H-Vimentin-R | ACCGTCTTAATCAGAAGTGTCCT |
| H-snail-R | CTCGGACCTTCTCCCGAATG |
| H-snail-R | TCATCAAAGTCCTGTGGGGC |
| H-α-SMA-F | CTATGAGGGCTATGCCTTGCC |
| H-α-SMA-R | GCTCAGCAGTAGTAACGAAGGA |
| H-MCL-1-F | ACTTCTCACTTCCGCTTCCT |
| H-MCL-1-R | TGAGTCCGATTACCGCGTTT |
| hsa-miR-29a-3p-F | TAGCACCATCTGAAATCGGTTA |
| hsa-miR-29a-3p-R | GCTGTCAACGATACGCTACGTA |
| hsa- miR-29b-3p-F | TAGCACCATTTGAAATCAGTGTT |
| hsa- miR-29b-3p-R | GCTGTCAACGATACGCTACGTA |
| hsa- miR-29c-3p-F | TAGCACCATTTGAAATCGGTTA |
| hsa- miR-29c-3p-R | GCTGTCAACGATACGCTACGTA |
| hsa-miR-181a-5p-F | CGCAACATTCAACGCTGTCGG |
| hsa-miR-181a-5p-R | GCTGTCAACGATACGCTACGTA |
| hsa-miR-181b-5p-F | AACATTCATTGCTGTCGGTGGGT |
| hsa-miR-181b-5p-R | GCTGTCAACGATACGCTACGTA |
