# Supplementary figures and images for: lncRNA CRNDE Affects Th17/IL-17A and Inhibits Epithelial-Mesenchymal Transition in Lung Epithelial Cells Reducing Asthma Signs
Source: Oxid Med Cell Longev. 2023 Jan 27;2023:2092184. doi: 10.1155/2023/2092184 (PMC9897922; doi:10.1155/2023/2092184)

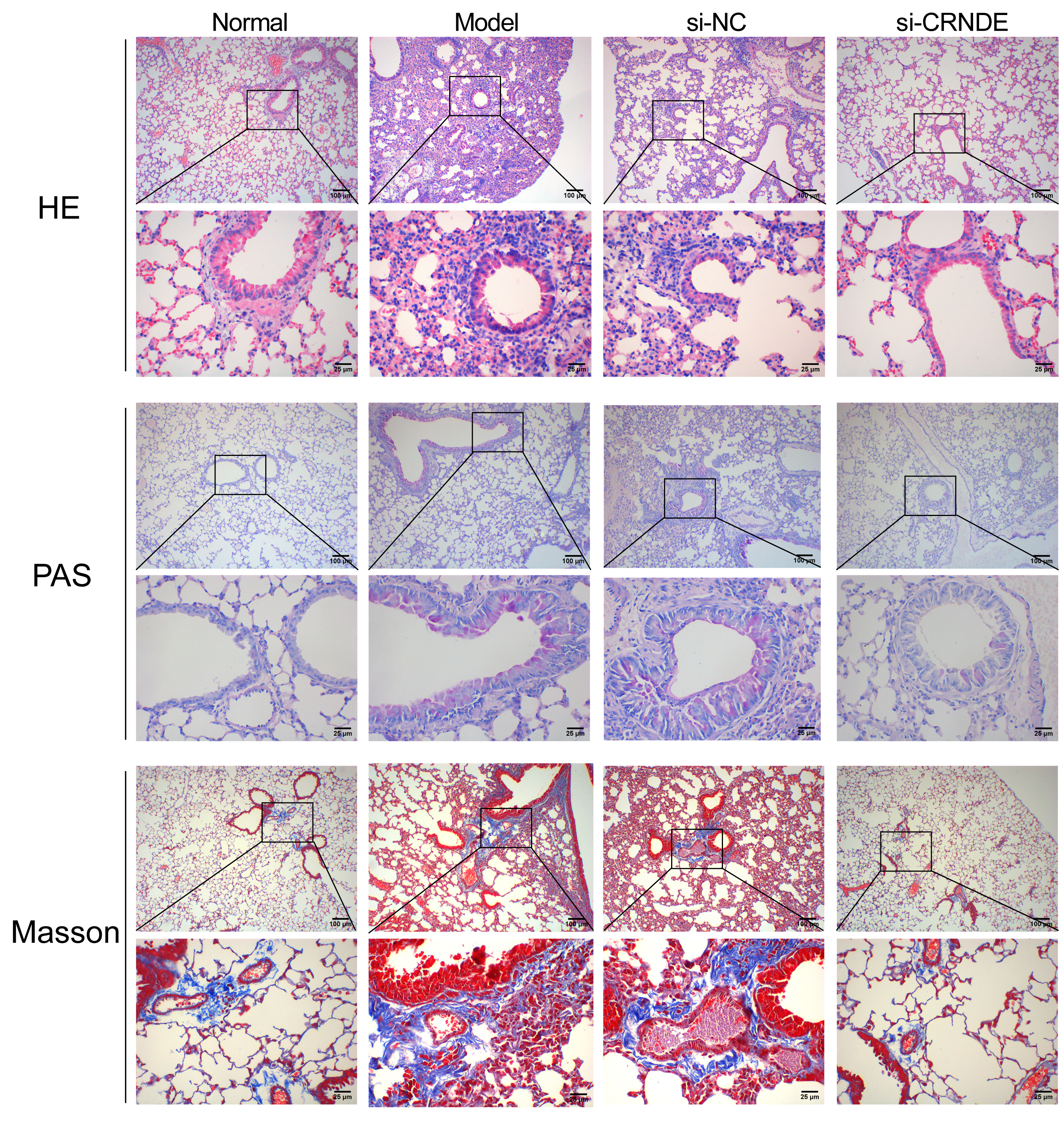

Supplement: Supplementary Materials — Supplementary Figure S1: HE, PAS, and Masson's stainings were applied to observe tissue morphologies. The model group samples displayed inflammatory cell infiltration; the number of PAS positive cells and the collagen fiber content were higher than those in the control group samples. These changes were reversed after transfection with si-CRNDE. Scale bar = 25/100 μm. Supplementary Figure S2: IF was applied to detect the distribution of E-cadherin and vimentin. Scale bar = 25 μm. ∗P < 0.05 vs. si-NC. #P < 0.05 vs. si-CRNDE+oe-NC. Supplement table 1: the information of antibody. Supplement table 2: the primer sequence. [file 2092184.f1.zip › Figure S1 (1).jpg]

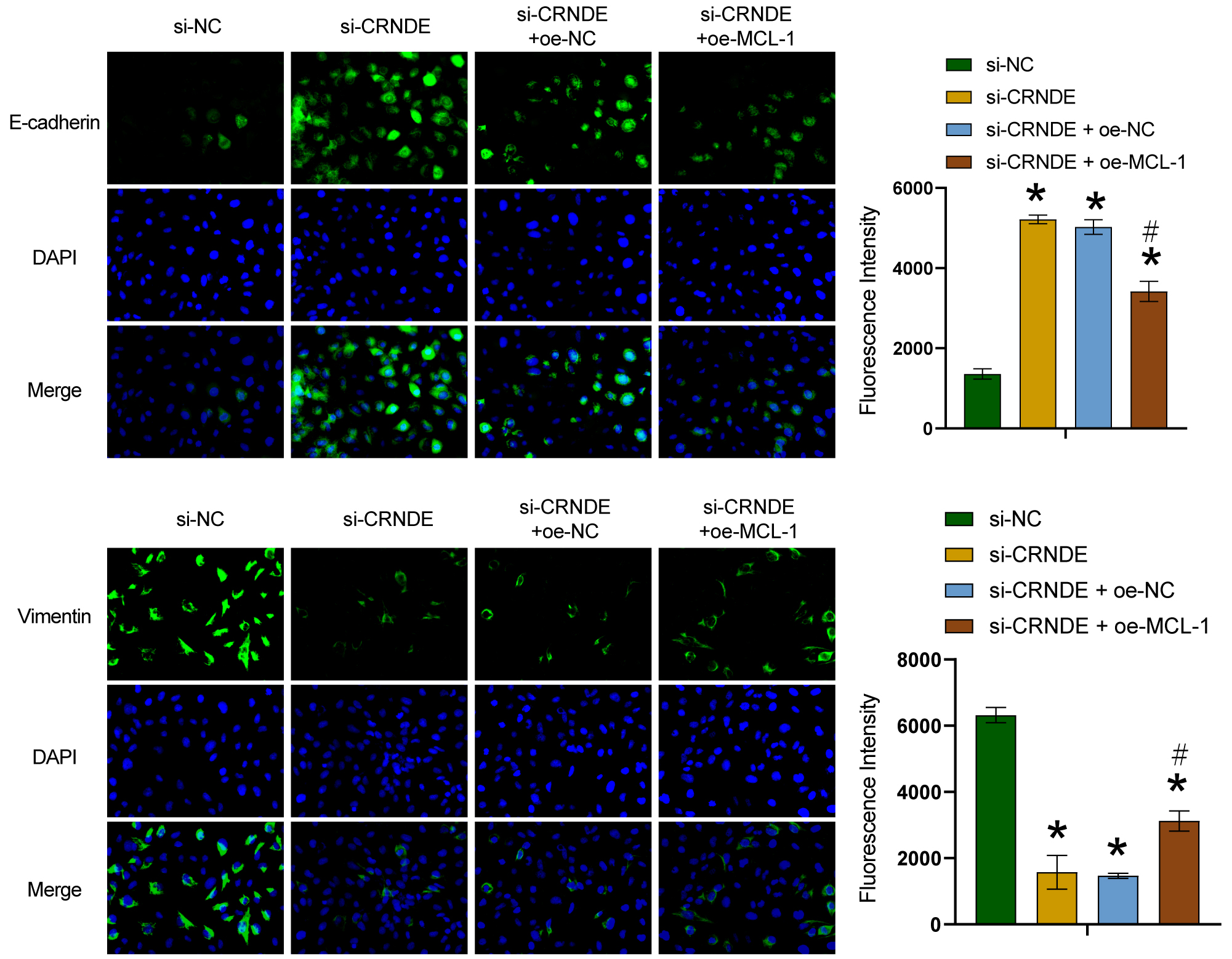

Supplement: Supplementary Materials — Supplementary Figure S1: HE, PAS, and Masson's stainings were applied to observe tissue morphologies. The model group samples displayed inflammatory cell infiltration; the number of PAS positive cells and the collagen fiber content were higher than those in the control group samples. These changes were reversed after transfection with si-CRNDE. Scale bar = 25/100 μm. Supplementary Figure S2: IF was applied to detect the distribution of E-cadherin and vimentin. Scale bar = 25 μm. ∗P < 0.05 vs. si-NC. #P < 0.05 vs. si-CRNDE+oe-NC. Supplement table 1: the information of antibody. Supplement table 2: the primer sequence. [file 2092184.f1.zip › Figure S2 (1).jpg]
